# Evaluation of Phenolic Compounds and Antioxidant and Antimicrobial Activities of Some Common Herbs

**DOI:** 10.1155/2017/3475738

**Published:** 2017-02-16

**Authors:** Muhammad Abdul Qadir, Syeda Kiran Shahzadi, Asad Bashir, Adil Munir, Shabnam Shahzad

**Affiliations:** ^1^Institute of Chemistry, University of the Punjab, Lahore 54590, Pakistan; ^2^Department of Chemistry, Minhaj University, Lahore 54590, Pakistan

## Abstract

The study was designed to evaluate the phenolic, flavonoid contents and antioxidant and antimicrobial activities of onion (*Allium cepa*), garlic (*Allium sativum*), mint (*Mentha spicata*), thyme (*Thymus vulgaris*), oak (*Quercus*), aloe vera (*Aloe barbadensis* Miller), and ginger (*Zingiber officinale*). All extracts showed a wide range of total phenolic contents, that is, 4.96 to 98.37 mg/100 g gallic acid equivalents, and total flavonoid contents, that is, 0.41 to 17.64 mg/100 g catechin equivalents. Antioxidant activity (AA) was determined by measuring reducing power, inhibition of peroxidation using linoleic acid system, and 2,2-diphenyl-1-picrylhydrazyl radical (DPPH) scavenging activity. Different extracts inhibited oxidation of linoleic acid by 16.6–84.2% while DPPH radical scavenging activity (IC_50_ values) ranged from 17.8% to 79.1 *μ*g/mL. Reducing power at 10 mg/mL extract concentration ranged from 0.11 to 0.84 nm. Furthermore the extracts of these medicinal herbs in 80% methanol, 80% ethanol, 80% acetone, and 100% water were screened for antimicrobial activity by disc diffusion method against selected bacterial strains,* Staphylococcus aureus*,* Escherichia coli*,* Bacillus subtilis*, and* Pasteurella multocida*, and fungal strains,* Aspergillus niger*,* Aspergillus flavus, Rhizopus solani*, and* Alternaria alternata*. The extracts show better antimicrobial activity against bacterial strains as compared to fungal strains. Results of various assays were analyzed statistically by applying appropriate statistical methods.

## 1. Introduction

In developing countries, 65%–80% of population depends upon herbal medicines for primary health care [1]. Different categories of bioactive compounds are being isolated and characterized since the middle of 19th century. Most of these compounds are used as raw material for new medicines or as an active ingredient of existing medicines. Herbal medicines provide rich amount of tannins, alkaloids, flavonoids, phenolic compounds, and so forth, so these can be used in the treatment of several degenerative disorders [2, 3].

Antioxidants play a vital role in free radical scavenging and chain breaking of oxidation reactions both in vivo and in vitro. Free radicals possess free unpaired electrons, making them highly unstable and can extract electron from other molecules to attain stability causing them damage. Among the potential uses of antioxidants, some are prevention of diseases related to oxidative stress in humans and also prevention of oxidative reactions in pharmaceuticals, cosmetic products, and food [4].

Utilization of synthetic antioxidants, that is, citric acid, propyl gallate, butylated hydroxyanisole (BHA), and butylated hydroxytoluene (BHT) in foods, leads to many side effects. For instance, these synthetic antioxidants have carcinogenic effect in living systems and many reports indicated that they may enhance microsomal enzyme activity and also enlarge liver size. Consequently, there is an increase interest in finding natural antioxidant agents capable of scavenging free radicals and hindering oxidative rancidity of lipids, in this way, protecting living organisms from diseases and retarding food spoilage [5].

Vegetables, grains, and fruits contain a huge variety of bioactive phytochemicals. The antioxidants which are derived from plants may function as free radicals scavengers, metal ion chelators, and reducing agents. It has been demonstrated that plasma antioxidant activity increases after consuming food high in antioxidants. That is why phytochemicals may fight against oxidative stress by maintaining a balance between antioxidants and oxidants. Many medicinal plants possess antioxidant properties. Antioxidants extracted from plants either in form of raw extracts or as their chemical constituents are very effective to stop the destructive processes caused by oxidative stress [6, 7].

Our aim was to evaluate the antioxidant, antibacterial, and antifungal activities, total flavonoid contents, and total phenolic contents of 6 commonly medicinal plants, onion (*Allium cepa*), garlic (*Allium sativum*), mint (*Mentha spicata*), thyme (*Thymus vulgaris*), oak (*Quercus*), aloe vera (*Aloe barbadensis* Miller), and ginger (*Zingiber officinale*). Antioxidant activity was determined by measuring inhibition of peroxidation using linoleic acid system, measuring reducing power and DPPH scavenging activity. Antimicrobial activity was determined by disc diffusion assay against four selected bacterial strains,* Pasteurella multocida*,* Staphylococcus aureus*,* Bacillus subtilis*, and* Escherichia coli*, and four fungal strains,* Alternaria alternata*,* Aspergillus niger, Rhizopus solani*, and* Aspergillus flavus*.

## 2. Materials and Methods

Samples of different medicinal plants were collected from local market of Lahore and Sialkot, Pakistan. All chemicals used throughout the study were of analytical grade. All readings were taken in triplicate and average results are presented.

### 2.1. Preparation of Plants Extracts

Dried samples were grounded to pass 80 mesh sieves. Each sample (20 g) was extracted with 200 mL of 80% ethanol, 80% methanol, 80% acetone, and 100% distilled water and was shaken for 1 day at room temperature followed by filtrating. Solvent was evaporated after concentrating the extracts at 65°C under reduced pressure, using rotary evaporator. Each dry extract was then weighed; yield was calculated and stored in at 4°C.

### 2.2. Determination of Total Phenolic Contents (TPC)

To determine the amount of total phenolic contents in plant extracts, Folin-Ciocalteu reagent was used [8]. Briefly, 0.5 mL of Folin-Ciocalteu reagent and 7.5 mL distilled water was added in 50 mg of crude plant extract and incubated for 10 minutes at room temperature. Afterwards, 20% Na_2_CO_3_ (1.5 mL) was added in the resulting mixture and heated at 40°C for 20 minutes. Absorbance was taken at 755 nm. The results were expressed as gallic acid equivalents mg/100 g of crude plant matter.

### 2.3. Determination of Total Flavonoid Contents (TFC)

To determine the amount of total flavonoid contents, procedure was used given at [8]. Briefly, 5 mL of distilled water and 0.1 g/mL of aqueous plant extracts were mixed together. After 5 minutes, 0.3 mL of 5% sodium nitrite and 0.6 mL of 10% aluminium chloride were added. 2 mL of 1 M sodium hydroxide was then added after another 5 minutes. At 510 nm, absorbance was recorded.

TFC were expressed as catechin equivalents per dry matter.

### 2.4. Evaluation of Antioxidant Activity of Extracts

Antioxidant activities of plants extracts were measured using following antioxidant assays.

#### 2.4.1. DPPH Scavenging Assay

DPPH scavenging assay was performed by method described by [9]. 0.2–500 *μ*g/mL of each extract was dissolved in 95% methanol and in 90 *μ*M DPPH solution and left for 60 minutes. Then OD was measured at 515 nm wavelength. Butylated hydroxyl toluene was employed as a standard. DPPH radical scavenging activity was calculated by following equation:

(1) $$\begin{array}{c} I\%=100-\frac{\left({Absorbance}_{blank}-{Absorbance}_{sample}\right)}{{Absorbance}_{blank}}. \end{array}$$

#### 2.4.2. Percent Inhibition in Linoleic Acid System

% inhibition of linoleic acid peroxidation was used to determine the antioxidant activities of plants extracts as described by [10]. 0.13 mL of linoleic acid solution, 0.2 M sodium phosphate buffer (pH 6.6), and 99.8% ethanol were mixed in 5 mg of each plant extract and incubated for 72 hours at 40°C. Colorimetric method was used to determine the extent of oxidation [11]. Briefly, ammonium thiocyanate solution (30% w/v), ethanol (75% v/v), ferrous chloride solution (20 mM in 3.5 HCl; v/v), and plant extracts were mixed under stirring. Absorbance was taken at 500 nm. Butylated hydroxytoluene was employed as positive control. Percent inhibition of linoleic acid oxidation was calculated using

(2) %  inhibition  of  linoleic  acid  oxidation=100−Abs.  increase  of  sample  at  175 hAbs.  increase  of  control  at  175 h×100.

#### 2.4.3. Reducing Power Assay

Antioxidant activities of plants extracts by reducing power assay was done using protocols described by [10], with some modifications. 5–10 mg of each dry concentrated extract was dissolved in 1% potassium ferricyanide and 0.2 M sodium phosphate buffer (pH 6.6). The resulting mixture was incubated for 20 minutes at 50°C. Afterwards, 10% trichloroacetic acid was added followed by centrifugation at 5°C. Upper layer was diluted with equal volume of deionized water and 0.1% ferric chloride. OD was measured at 700 nm.

### 2.5. Antimicrobial Activity

The antimicrobial activities of medicinal plants extracts were determined using disc diffusion method. 100 *μ*L of suspension, having 10^4^ CFU/mL of fungal spores and 10^8^ CFU/mL of bacterial strains, was dispensed on potato dextrose agar and nutrient agar medium, respectively.

Filter discs were individually impregnated with 30 *μ*L (3 mg/disc) of extract (100 mg/mL, 90 *μ*g/disk) and placed on the previously inoculated agar with chosen microorganism. As positive control/standard, Rifampicin was used for bacterial strains. For fungal strains, Terbinafine was used. Petri plates were incubated at 4°C for 60 minutes. Then the petri plates were kept for 48 hours at 30°C for fungal spores and at 37°C for 1 day for bacteria. Antimicrobial activities were determined by determining growth inhibition zones diameter in millimeters (including 6 mm disc diameter) against the selected organisms and comparing results with the controls.

### 2.6. Statistical Analysis

The data is presented as mean value ± SD value. One-way ANOVA procedure was used to perform the analysis of variance. Minitab software was used to calculate the significant differences (*p* < 0.05) between mean values.

## 3. Results and Discussion

Recently there is an increasing interest among the food researchers to distinguish antimicrobial compounds and antioxidants that have natural origin and are safe to use. Numerous flavors and herbs are accounted for to be a suitable source of antimicrobial and antioxidant agents. Over recent years, a number of studies have demonstrated that polyphenols that are present in dietary and herbal products hinder oxidative stress.

Preventive part of these foods is because of their components, particularly anthocyanidins, polyphenolics, flavonoids, and anthocyanins. Present research study was directed to assess the antioxidants, total phenolic contents, total flavonoid contents, and antimicrobial activities of extracts of garlic, onion, ginger, mint, aloe vera, thyme, and oak. Plant materials were extracted by using one extraction method, that is, orbital shaking, and four solvents, that is, 80% ethanol, 80% acetone, 80% methanol, and 100% water.

% yield (mg/100 g) of all plants extracts was within the range of 2.9 to 12.5 mg/100 g. Maximum yield (12.5 mg) was observed with 80% ethanolic extract of garlic. With regard to solvent efficacy, 80% ethanol was discovered more effective for the recovery of antioxidants from herbal plants.

**Figure 1:**
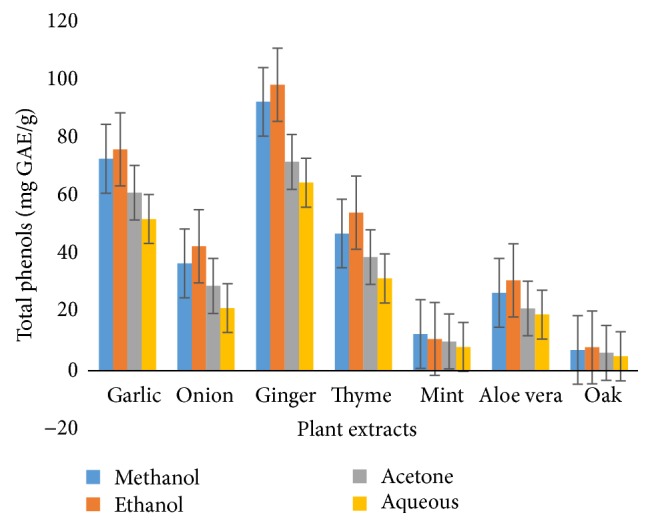
Total phenolic contents.

Total phenolic contents of medicinal plants extracts obtained from four different solvent systems vary from 98.37 mg GAE/100 g to 4.96 mg GAE/100 g (Figure 1). However total phenolic content of 80% ethanolic ginger extract was observed to be maximum (98.37 mg GAE/100 g) and that of aqueous extract of oak was the lowest (4.96 mg GAE/100 g).

**Figure 2:**
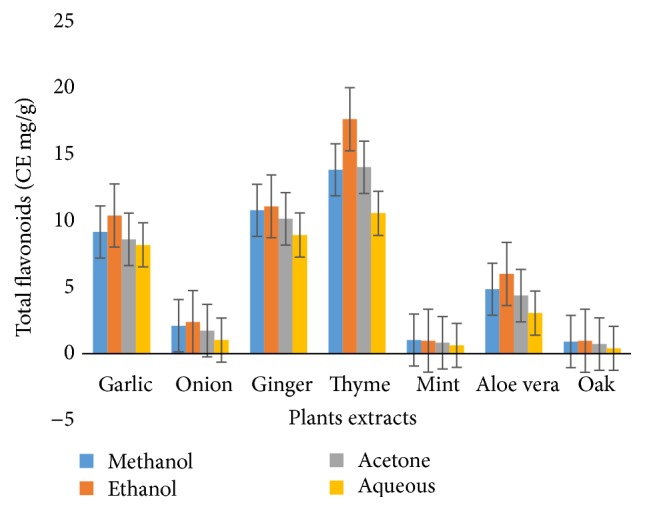
Total flavonoid Contents.

The total flavonoid contents of plant extracts obtained from four solvent systems ranged from 0.41 to 17.64 mg CE/100 g (Figure 2). The value of total flavonoids was observed to be highest, that is, 17.64 mg CE/100 g obtained from 80% ethanolic garlic extract, while the minimum value was 0.41 mg CE/100 g for aqueous oak extracts.

**Figure 3:**
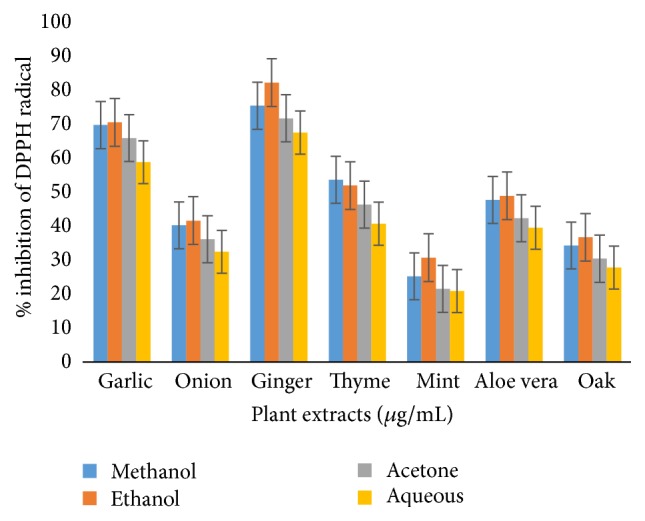
DPPH radical scavenging activity (IC_50_) of extracts from different medicinal plants.

Antioxidant activity as determined by DPPH assay was found to be maximum in 80% ethanolic ginger extract (82.2%) and minimum by aqueous extract of mint (20.9%) (Figure 3). IC_50_ values, which represented the concentration of antioxidants that caused 50% neutralization of DPPH radicals, were calculated from the plot of inhibition percentage against concentration.

DPPH radical scavenging activity (IC_50_ values) ranged from 17.8% to 79.1 *μ*g/mL (Table 1).

**Figure 4:**
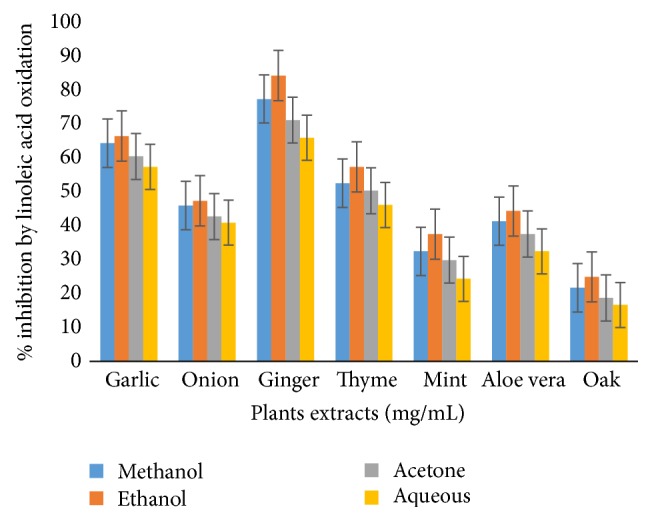
Percentage inhibition of linoleic acid oxidation.

The ethanolic extract of garlic was found to demonstrate maximum inhibition of peroxidation, reflecting highest antioxidant activity, while least inhibition was seen by oaks. With regard to solvents, 80% ethanol was discovered more proficient for the recovery of antioxidant compounds from medicinal herbs as compared to other solvent systems (Figure 4).

With the increase of antioxidant compounds in tested medicinal plants, there was an observed increase in reducing power. Each plant extract displayed a dose-dependent reducing power (shown as absorbance at 700 nm) within range of 2.5–10 mg of extracts per mL. Reducing power of ethanolic plant extracts was found to be highest while that of aqueous was found to be the lowest as shown in Table 2. Extraction efficiency of components with antioxidative properties was lowering in the following order: ginger > garlic > onion > thyme > aloe vera > mint > oaks.

The antimicrobial activity of all plants extracts was evaluated against four pathogenic bacterial strains (*Bacillus subtilis*,* Staphylococcus aureus*,* Pasteurella multocida*, and* Escherichia coli*) and four fungal strains (*Aspergillus flavus*,* Rhizopus solani, Aspergillus niger*, and* Alternaria alternate*). Antimicrobial potential of herbal plants extracts was assessed in terms of zone of inhibition of microbe's growth as shown in Table 3. All extracts showed better antibacterial activity as compared to antifungal activity. Results revealed that ethanolic and methanolic plant extracts were more active against microbes as in comparison with acetone and aqueous extracts. Of all the plants extracts, ginger and garlic show better antibacterial and antifungal activity than others.

## 4. Conclusion

Results of our study show that tested plants, onion (*Allium cepa*), garlic (*Allium sativum*), mint (*Mentha spicata*), thyme (*Thymus vulgaris*), oak (*Quercus*), aloe vera (*Aloe barbadensis* Miller), and ginger (*Zingiber officinale*), are potent source of antioxidants. These plants extracts also showed good antibacterial and antifungal activities against pathogenic microbes which suggest that these plants could be used to treat various infections caused by microbes.

## Figures and Tables

**Table 1:** DPPH radical scavenging activity (IC_50_) of extracts from different medicinal plants.

Plants	Methanol	Ethanol	Acetone	Aqueous
Garlic	30.3	29.5	34.1	41.2
Onion	59.8	58.4	63.9	67.6
Ginger	24.6	17.8	28.3	32.5
Thyme	46.4	48.1	53.7	59.3
Mint	74.8	69.3	78.5	79.1
Aloe vera	52.3	51.1	57.7	60.5
Oak	65.7	63.3	69.6	71.2

Values (mean ± SD) of extracts, analyzed individually in triplicate.

**Table 2:** Reducing power (in terms of absorbance values at 700 nm) of extracts from different medicinal plants.

Plants	Conc. (mg/mL)	Methanol	Ethanol	Acetone	Aqueous
		Absorbance at 700 nm			
Garlic	2.5	0.19 ± 0.02	0.21 ± 0.05	0.12 ± 0.02	0.09 ± 0.02
	5	0.39 ± 0.04	0.44 ± 0.03	0.26 ± 0.04	0.16 ± 0.04
	7.5	0.61 ± 0.03	0.67 ± 0.04	0.38 ± 0.04	0.26 ± 0.02
	10	0.82 ± 0.05	0.84 ± 0.06	0.51 ± 0.06	0.34 ± 0.05

Onion	2.5	0.14 ± 0.02	0.16 ± 0.02	0.10 ± 0.03	0.06 ± 0.03
	5	0.29 ± 0.04	0.33 ± 0.03	0.19 ± 0.02	0.10 ± 0.05
	7.5	0.45 ± 0.04	0.50 ± 0.02	0.31 ± 0.04	0.17 ± 0.03
	10	0.61 ± 0.05	0.63 ± 0.05	0.42 ± 0.03	0.25 ± 0.06

Ginger	2.5	0.21 ± 0.03	0.23 ± 0.04	0.17 ± 0.02	0.12 ± 0.02
	5	0.40 ± 0.04	0.45 ± 0.03	0.33 ± 0.04	0.25 ± 0.04
	7.5	0.62 ± .08	0.69 ± 0.02	0.50 ± 0.04	0.37 ± 0.03
	10	0.84 ± 0.04	0.88 ± 0.05	0.64 ± 0.06	0.49 ± 0.05

Thyme	2.5	0.18 ± 0.03	0.2 ± 0.02	0.13 ± 0.03	0.09 ± 0.03
	5	0.32 ± 0.04	0.41 ± 0.03	0.27 ± 0.02	0.19 ± 0.02
	7.5	0.51 ± 0.02	0.59 ± 0.04	0.41 ± 0.04	0.28 ± 0.04
	10	0.68 ± 0.03	0.76 ± 0.02	0.56 ± 0.05	0.37 ± 0.09

Mint	2.5	0.08 ± 0.02	0.1 ± 0.02	0.06 ± 0.03	0.03 ± 0.03
	5	0.17 ± 0.04	0.19 ± 0.03	0.13 ± 0.02	0.07 ± 0.05
	7.5	0.26 ± 0.04	0.31 ± 0.02	0.2 ± 0.04	0.11 ± 0.03
	10	0.34 ± 0.05	0.38 ± 0.05	0.25 ± 0.03	0.14 ± 0.06

Aloe vera	2.5	0.15 ± 0.03	0.17 ± 0.04	0.12 ± 0.02	0.08 ± 0.02
	5	0.29 ± 0.04	0.35 ± 0.03	0.25 ± 0.04	0.19 ± 0.04
	7.5	0.42 ± 0.07	0.5 ± 0.02	0.37 ± 0.04	0.28 ± 0.03
	10	0.58 ± 0.02	0.66 ± 0.02	0.51 ± 0.03	0.36 ± 0.02

Oak	2.5	0.05 ± 0.02	0.07 ± 0.02	0.02 ± 0.03	0.02 ± 0.03
	5	0.11 ± 0.04	0.15 ± 0.03	0.05 ± 0.02	0.04 ± 0.05
	7.5	0.16 ± 0.04	0.22 ± 0.02	0.09 ± 0.04	0.08 ± 0.03
	10	0.21 ± 0.05	0.27 ± 0.05	0.13 ± 0.03	0.11 ± 0.06

Values are the mean ± standard deviation of triplicate.

**Table 3:** Antimicrobial activities in terms of inhibition zones (mm) of plants extracts in different solvents.

Plants	Bacterial strains	Methanol	Ethanol	Acetone	Aqueous	Rifampicin	Fungal strains	Methanol	Ethanol	Acetone	Aqueous	Terbinafine
		Inhibition zones (mm)										
Garlic	*E. coli*	10.3	11.7	8.4	7.5	21.7	*A. niger*	12.3	12.5	10.6	9	19.9
	*P. multocida*	13.7	14.4	11.1	9.3	25.4	*A. flavus*	9.9	10.3	8.1	7.4	20.6
	*B. subtilis*	11.1	11.5	9.7	7.9	23.1	*R. solani*	10.4	9.7	7.7	6.8	23.1
	*S. aureus*	14.4	15.3	12.4	10.6	28.2	*A. alternata*	12.6	12.1	9.1	7.2	21.7

Onion	*E. coli*	9.4	11.5	8.8	6.2	21.7	*A. niger*	11.2	11.5	12.5	11.7	19.9
	*P. multocida*	12.7	13.6	11.1	9.3	25.4	*A. flavus*	11.9	11.4	12.1	12.5	20.6
	*B. subtilis*	11.7	12.3	10.1	8.3	23.1	*R. solani*	10.1	9.3	10.2	8.6	23.1
	*S. aureus*	12.4	11.7	12.4	7.5	28.2	*A. alternata*	12.6	12.8	9.7	10.3	21.7

Ginger	*E. coli*	11.4	11.8	9.1	7.7	21.7	*A. niger*	12.4	12.2	10.5	8.3	19.9
	*P. multocida*	12.6	13.2	10.3	8.1	25.4	*A. flavus*	9.1	9.4	7.6	10.4	20.6
	*B. subtilis*	13.1	14.6	11.4	10.4	23.1	*R. solani*	10.1	10.5	11.5	9.6	23.1
	*S. aureus*	15.2	15.7	12.4	12.1	28.2	*A. alternata*	14.5	14.9	13.1	11.7	21.7

Thy	*E. coli*	9.5	10.1	—	—	21.7	*A. niger*	10.5	9.7	—	—	19.9
	*P. multocida*	12.3	12.7	8.9	—	25.4	*A. flavus*	6.9	6.6	—	—	20.6
	*B. subtilis*	7.1	8.2	6.4	—	23.1	*R. solani*	12.4	12.9	7.6	—	23.1
	*S. aureus*	14.6	15.1	10.5	—	28.2	*A. alternata*	10.1	10.7	—	—	21.7

Mint	*E. coli*	7.5	8.4	—	—	21.7	*A. niger*	9.2	9.4	10.7	6.4	19.9
	*P. multocida*	10.1	12.4	10.5	8.4	25.4	*A. flavus*	6.8	7.2	8.4	—	20.6
	*B. subtilis*	8.9	11.3	—	—	23.1	*R. solani*	10.2	9.5	6.3	—	23.1
	*S. aureus*	14.6	15.1	10.7	6.8	28.2	*A. alternata*	13.7	12.9	10.1	8.3	21.7

Aloe vera	*E. coli*	11.5	13.9	—	—	21.7	*A. niger*	12.3	10	10.7	—	19.9
	*P. multocida*	13.3	14.1	8.5	—	25.4	*A. flavus*	14	14.3	11.5	—	20.6
	*B. subtilis*	10.7	8.4	—	—	23.1	*R. solani*	9.7	10.4	9.1	—	23.1
	*S. aureus*	8.5	7.9	11.5	—	28.2	*A. alternata*	12.7	13.2	10.9	—	21.7

Oak	*E. coli*	8.9	9.3	10	12.7	21.7	*A. niger*	8	7.8	12.1	11.9	19.9
	*P. multocida*	12.2	11.7	13.5	14.1	25.4	*A. flavus*	11.9	12.4	11.4	12.7	20.6
	*B. subtilis*	7.7	8	9.8	10.5	23.1	*R. solani*	8.3	8.6	9.7	10.6	23.1
	*S. aureus*	11.3	12.6	14.4	15.2	28.2	*A. alternata*	11.5	11.7	12.3	12.9	21.7

Values are the mean ± standard deviation of triplicate.
